# Resting State Networks' Corticotopy: The Dual Intertwined Rings Architecture

**DOI:** 10.1371/journal.pone.0067444

**Published:** 2013-07-24

**Authors:** Salma Mesmoudi, Vincent Perlbarg, David Rudrauf, Arnaud Messe, Basile Pinsard, Dominique Hasboun, Claudia Cioli, Guillaume Marrelec, Roberto Toro, Habib Benali, Yves Burnod

**Affiliations:** 1 UMR-S 678, Laboratoire d'Imagerie Fonctionnelle, Inserm Univ. Pierre et Marie Curie, Paris 6, Paris, France; 2 Univ. Paris 1, MATRICE Program, Paris, France; 3 CENIR, Institut du Cerveau et de la Moelle épiniere, Hôpital Pitié-Salpêtrière, Paris, France; 4 ICM-Institut du Cerveau et de la Moelle épiniere, Hôpital Pitié-Salpêtrière, Paris, France; 5 UMR-S 975, INSERM, Paris, France; 6 UMR 7225, CNRS, Univ. Pierre et Marie Curie, Hôpital Pitié-Salpêtrière, Paris, France; 7 Human Genetics and Cognitive Functions, Institut Pasteur, Paris, France; 8 CNRS URA 2182 “Genes, synapses and cognition”, Institut Pasteur, Paris, France; 9 Univ. Paris Diderot, Sorbonne Paris Cité, Human Genetics and Cognitive Functions, Paris, France; Indiana University, United States of America

## Abstract

How does the brain integrate multiple sources of information to support normal sensorimotor and cognitive functions? To investigate this question we present an overall brain architecture (called “the dual intertwined rings architecture”) that relates the functional specialization of cortical networks to their spatial distribution over the cerebral cortex (or “corticotopy”). Recent results suggest that the resting state networks (RSNs) are organized into two large families: 1) a sensorimotor family that includes visual, somatic, and auditory areas and 2) a large association family that comprises parietal, temporal, and frontal regions and also includes the default mode network. We used two large databases of resting state fMRI data, from which we extracted 32 robust RSNs. We estimated: (1) the RSN functional roles by using a projection of the results on task based networks (TBNs) as referenced in large databases of fMRI activation studies; and (2) relationship of the RSNs with the Brodmann Areas. In both classifications, the 32 RSNs are organized into a remarkable architecture of two intertwined rings per hemisphere and so four rings linked by homotopic connections. The first ring forms a continuous ensemble and includes visual, somatic, and auditory cortices, with interspersed bimodal cortices (auditory-visual, visual-somatic and auditory-somatic, abbreviated as VSA ring). The second ring integrates distant parietal, temporal and frontal regions (PTF ring) through a network of association fiber tracts which closes the ring anatomically and ensures a functional continuity within the ring. The PTF ring relates association cortices specialized in attention, language and working memory, to the networks involved in motivation and biological regulation and rhythms. This “dual intertwined architecture” suggests a dual integrative process: the VSA ring performs fast real-time multimodal integration of sensorimotor information whereas the PTF ring performs multi-temporal integration (i.e., relates past, present, and future representations at different temporal scales).

## Introduction

To perform sensorimotor and cognitive functions, the brain needs to coordinate and integrate the activity of different regions that process multiple sources of information [1]–[5]. This integration is based on correlated activities that can be seen, for example, in the coherent brain networks revealed by resting-state magnetic functional imaging [6], [7].

Recent results suggest that these resting state networks (RSNs) are organized according to some general anatomo-functional principles [8]–[14]. First, RSNs appear to be similar to the Task-Based Networks (TBNs) that emerge from the analysis of large ensembles of activation studies collected with different cognitive tasks found in very large functional imaging databases [13], [14]. These TBNs can be used as a reference set to infer the sensorimotor and cognitive functions of cortical networks revealed by RSNs. Second, several studies have shown that RSNs can be grouped into two major families, a “sensorimotor family” and an “association family,” that rely on two different modes of integration [8]–[12], [15]. In the “sensorimotor family,” the RSNs encompass a set of topographically adjacent Brodmann Areas (BAs), that includes the processing hierarchies of primary and secondary unimodal areas, and bimodal sensorimotor areas. In the “association family,” the RSNs encompass a topographically discontinuous set of BAs distributed over temporal, parietal, and frontal areas characterized by strong functional coupling between distant regions. These association networks correspond to the RSNs, previously described as the (1) default mode, (2) dorsal attentional, (3) ventral attentional, and (4) frontoparietal control networks (see, e.g., [16]–[22]). Similar results have been obtained using different RSNs extraction and clustering methods, either topographical (local vs. large scale functional connectivity [8]), hierarchical [12], or based on stepwise connectivity [9].

Here, we further investigate the relationship between the functional and anatomical organizations of the RSNs. Several previous studies have already described various RSNs clusters obtained from direct temporal correlations between RSNs, a pattern suggesting global organizational principles, but the topographies of the RSNs on the cortical surface depend on the methods and parameters used (e.g., the preselected number of components in an ICA analysis [8], [12]).

For this reason, we chose to characterize anatomo-functional clusters of RSNs from their overlap with two reference sets [i.e., Brodmann Areas (BAs) and TBNs] to confirm the two principles suggested by the RSN literature: (1) RSNs connect adjacent BAs in sensorimotor regions or connect distant areas in associative regions specialized in higher cognitive functions (e.g., language, attention, working memory) and (2) RSNs resemble TBNs and their specialization can be inferred from the closest TBNs. Furthermore, we expected to find correlations between RSNs groups overlapping TBNs functional groups and RSNs groups overlapping groups of BAs, which are either adjacent (local connectivity) or extend over different brain regions.

In an initial step, we used two different groups of subjects, and for each group we automatically determined the number of RSNs, on the basis of their representativeness. This method was robust because it produced two similar sets of 32 RSNs, with greater representativeness in two independent groups of subjects. Then, we inferred the RSNs functional roles from the overlap of the RSNs with 18 reference TBNs extracted from large databases of fMRI activations obtained during sensorimotor and cognitive tasks [14]. Similarly, we characterized the RSNs topography and topological features from their overlap with the Brodman Areas, in order to determine the RSNs spanning adjacent cortical regions (e.g., BA17 and BA18), as well as those extending to distant regions (e.g., between the BA39 parietal area and the BA46 frontal area). We then extracted functional groups of RSNs from the RSN×TBN matrix and independently extracted anatomical groups from the RSN×BA matrix, using general classification methods (such as expectation maximization, see Method section). As an advantage of this method, these two well-defined reference sets (i.e., BAs and TBNs) are characterized independently of the RSNs extraction.

Remarkably, RSNs clusters having the same topography as sensorimotor and bimodal TBNs are found on adjacent BAs clusters. In turn, the RSNs, that have the same topography as the TBNs specialized in higher cognitive functions, emotions and biological regulations, are organized on networks linking distant parietal, frontal, temporal, and cingulate regions (PTF family). Our results showed that these two sets of cortical networks have a remarkable architecture that comprises two intertwined rings per hemisphere and so four rings linked by homotopic connections, an architecture that we called “the dual intertwined rings architecture.”

The *first* of these intertwined rings in each hemisphere is continuous over the cortex, organized in a concentric manner around the inferior parietal cortex, and relates visual, auditory, somatosensory and motor cortices with interspersed bimodal regions. This circular organization optimally integrates these sensorimotor modalities. We call this ring: the visual-sensorimotor-auditory (VSA) ring. The *second* ring in each hemisphere relates parietal, temporal, and frontal regions dedicated to higher cognitive functions (e.g., language, episodic memory, social interactions, self) with systems dedicated to emotions, basic biological regulations (e.g., hunger, thirst) and biological rhythms. We call this ring the Parieto Temporo Frontal (PTF) ring.

We called these two rings “*intertwined*” because the parietal region of the PTF ring (BAs 39 and 40) is at the center of the VSA ring and the PTF ring does not form a continuum over the cerebral cortex, but is closed through long-range association fiber tracts [23]. Taking into account the variety of sensorimotor and cognitive functions within each ring, the dual architecture of the rings suggests that each set of functions shares common basic neural principles. We hypothesize that the networks forming the two rings implement two different temporal integration processes: real-time integration of sensory and motor interactions with the world in the VSA ring and what we refer to as “multi-temporal integration” in the PTF ring, (i.e., the construction of interacting past, present and future representations, at different temporal scales).

## Results

### Extraction of the most representative RSNs in the group: robustness

**Figure 1:**
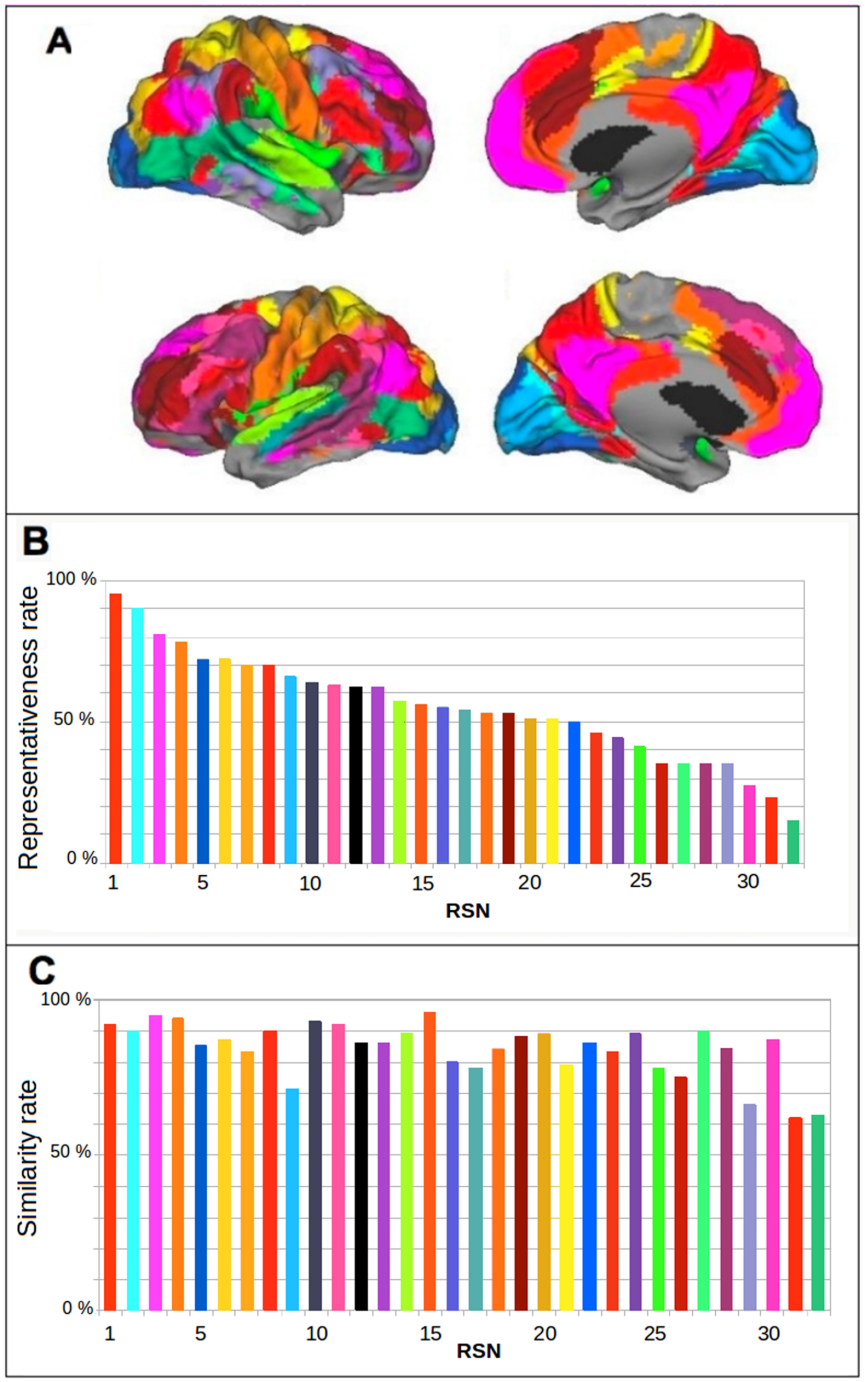
Mapping and representativeness of RSNs and similarities in two different populations. (A) Mapping of the 32 Resting State Networks (RSNs), on the right hemisphere (above) and the left hemisphere (below), on the lateral face (left) and the medial face (right). (B) Representativeness of the 32 RSNs. The color of each bar corresponds to the color of the RSNs network in Figure 1A and the RSNs are labeled with their representativeness rank. (C) Spatial similarity rate between equivalent RSNs in the Cambridge and Beijing populations.

We used two independent databases of resting state activity: the first was from a population of subjects from Cambridge (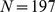) and the second was from a population of subjects from Beijing (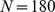). We started with the Cambridge database. We first applied spatially independent components analysis (sICA) to the voxel time series across each subject's brain. This provided one voxel factor scores matrix per subject. The matrices of all subjects were then concatenated into a group factor score matrix that was used in a hierarchical cluster analysis. From this analysis, we obtained 264 classes of voxels from which we selected 32 representative classes as the 32 most representative RSNs. These RSNs were observed in at least 10% of the subjects (see Fig. 1A and Fig. 1B). The first 20 classes were observed in at least 50% of the subjects (Fig. 1B). We labeled each RSN with its representativeness rank in the population (Fig. 1B). We then used the same general method with the independent database of subjects from Beijing. This analysis also yielded 32 RSNs. The RSNs found in the Cambridge database were very similar to those found in the Beijing database (Fig. 1C). The similarity rate was as high as 60%, showing that the 32 identified RSNs were highly representative and robust within and between independent populations.

### RSNs Specialization: overlap between RSNs and TBNs

**Figure 2:**
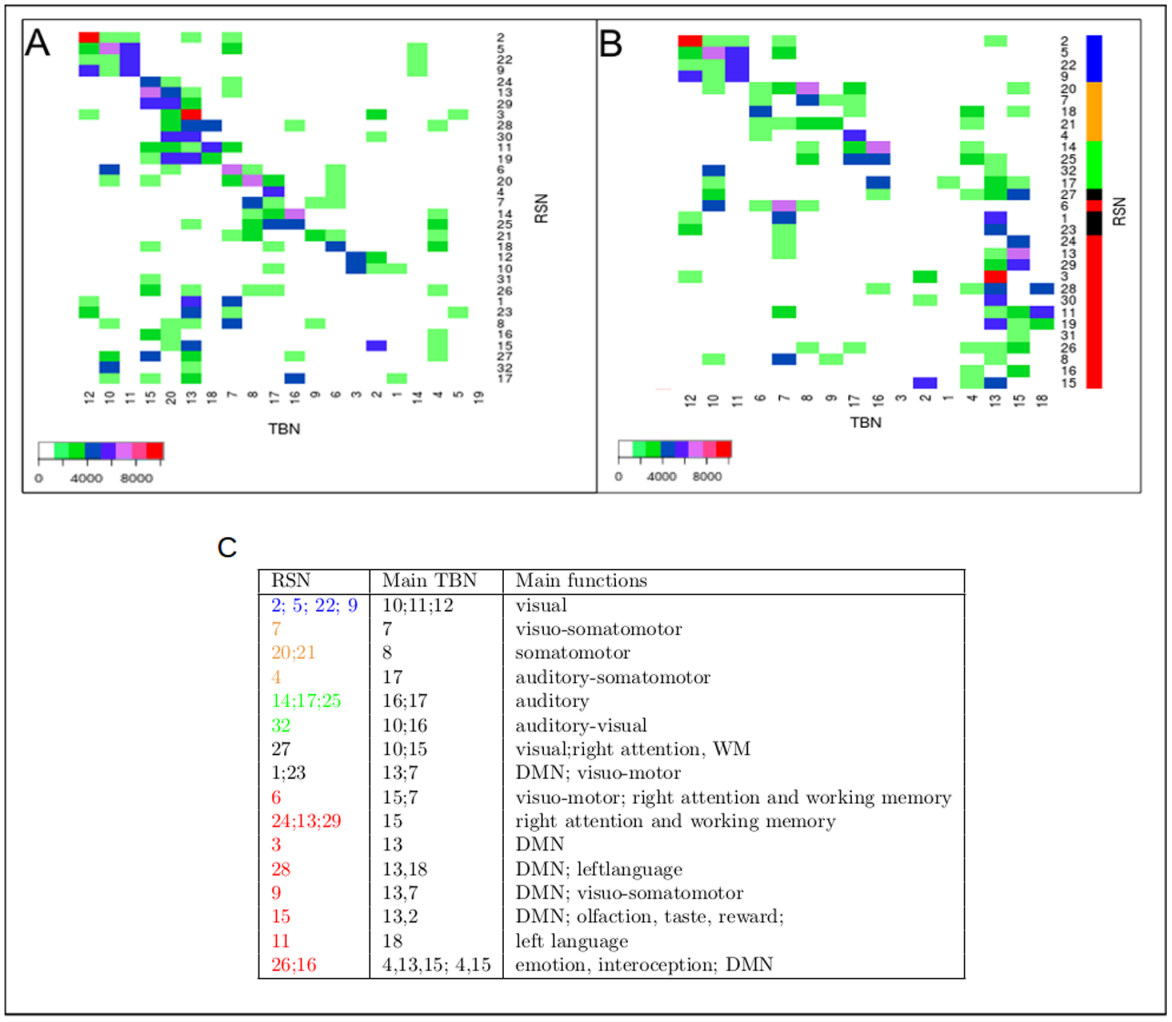
RSN×TBN overlap matrix. (A) RSN×TBN matrix showing overlaps (topographical similarity) between the 32 Resting State Networks (RSN, vertical axis) and the 18 reference Task Based Networks (TBNs, horizontal axis). The RSN are labeled as in Fig. 1 and the TBNs are labeled as in [14]. The RSNs and TBNs are ranked according to the maximum of their overlaps. (B) RSN×TBN matrix reorganized by applying an Expectation Maximization (EM) algorithm to display the RSN clusters having similar overlaps with the TBNs (see text for details). The RSN×TBN clusters are: 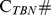 1 (RSN# 2, 5, 22, 9), 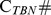 2 (20, 7, 18, 21), 
3 (4), 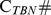
4 (14, 25), 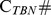
5 (32, 17, 27) and 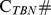
6 (6, 1, 23, 24, 13, 29, 3, 28, 30, 11, 19, 31, 26, 8, 16, 15). The color bars (at the right of Figure 2B) indicate: visual (blue), somatomotor (orange), auditory (green), left, right and bilateral RSN (red), and RSNs of intermediate region (black). Scale bars represents the nuber of shared voxels. (C) This table shows the correspondence between RSNs and TBNs obtained by the maximal overlap between them, with the main sensory-motor and cognitive functions of the TBNs. The masks, labels and functions of TBNs are taken from [14]. The labels of RSNs correspond to their representativeness in the group of subjects, as in Fig. 1. The colors used for RSNs labels, are the same as color bars in Fig. 2B.

Previous results have shown that RSNs are similar to TBNs extracted from a large database of activation studies across a variety of behavioral tasks [13], [14]. A systematic statistical analysis of thousands of datasets of brain activation scans obtained during behavioral tasks (sensory, motor, cognitive, and emotional) has previously revealed the existence of clusters of activation corresponding to TBNs (available on-line at www.brainmap.org/). We computed the overlaps between our 32 RSNs and the 18 reference TBNs (Fig. 2A).

The RSNs are represented along the vertical axis of the matrix and sorted according to their maximum overlap with the TBNs. The RSN×TBN matrix revealed a dual organization. The upper part of this matrix is nearly diagonal and reveals RSNs related to TBNs specialized in sensorimotor and cognitive functions (see Table in Fig. 2C). The lower part of the matrix reveals RSNs that correspond mainly to the Default Mode Network (“DMN,” TBN# 13, involved in social interactions) but also to several TBNs such as TBN number 15 (working memory and attention), 18 (language), 4 (emotion and interoception), and 2 (olfaction, taste, and reward). Since the TBNs database did not describe the specialization of subcortical structures, we focused on the cerebral cortex: RSN#10 and 12 corresponding to sub-cortical networks were eliminated from subsequent analyses. In order to further categorize the major RSNs families according to their overlap with TBNs, we used a clustering method based on mixture distributions (see Methods section). Fig. 2B shows the RSN×TBN matrix, in which RSNs along the vertical axis are sorted on the basis of the afore mentioned clustering method. The 18 TBNs on the horizontal axis of the TBN×RSN matrix were based on a meta-analysis of cognitive brain-imaging articles [14] (the TBNs were labeled “ICN” in this reference). For further details concerning the correspondence between RSNs and TBNs, see Table in Fig. 2C.

Six RSNs clusters based on TBNs overlap were identified (see Fig. 2B). Cluster 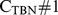 matches visual areas; cluster 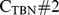 and 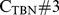 somatomotor regions; clusters 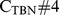 and 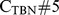 the auditory regions, and cluster 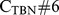 mainly large association networks (TBN# 18, 15, 13, 4, 1, 2 and 7). 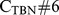 corresponds to four major functional entities: (1) for language (TBN# 18) in the left hemisphere; (2) for attention and working memory in the right hemisphere (TBN# 15); (3) for social interaction, self, memory recall and anticipation (TBN# 13) in a bilateral network corresponding to DMN; (4) for basic biological regulation, olfaction, taste and emotion (TBN# 2, 4).

These clustering results were confirmed by another method [24] (see Method section), with a similar significance level (i.e., 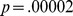).

#### RSNs topography: overlap between RSNs and the Brodmann Areas

**Figure 3:**
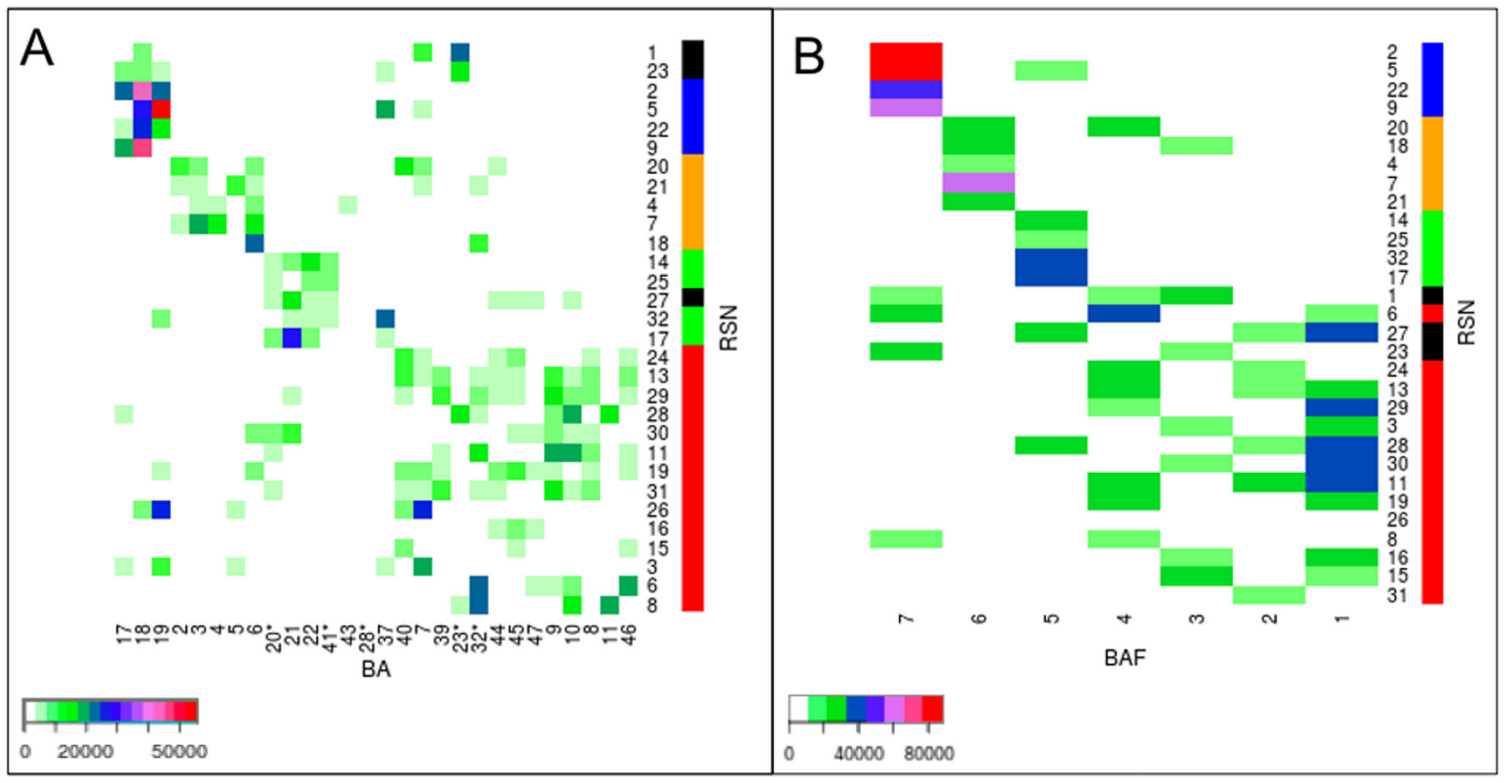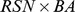 overlap matrix. (A) Matrix of 30RSN×28BA (Brodmann Area). 20*: BA20+BA38, 41*: BA41+42, 28*: BA28+BA34+BA35+BA36, 32*:BA32+BA24+BA25, 23*:BA23+BA29+BA30+BA31, (B) Matrix of 30RSN×7BAF (Brodmann Area Family), see the details concerning the BAFs and RSNs in the text. Both matrices are reorganized by applying the same Expectation Maximization (EM) algorithm as in Fig. 2C, to reveal the RSNs clusters with a similar topography on the cortical surface. The RSNs are ranked on the vertical axis according to this clustering. BA×RSN matrix clusters: 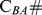
1 (RSN# 1, 23, 2, 5, 22, 9), 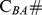
2 (20, 21), 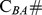
3 (4, 7, 18), 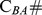
4 (14, 25, 27, 32,17), 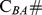
5 (24, 13, 29, 28, 30, 11, 19, 31, 26, 16), 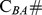
6 (3, 15) and 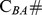
7 (6, 8). 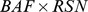 matrix clusters: 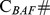
1 (RSN# 2, 22, 9, 5), 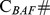
2 (4,7,21), 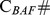
3 (18), 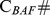
4 (20), 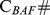
5 (25, 14, 17, 32), 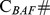
6 (27, 28, 11, 29, 13, 19, 6, 26, 18, 20, 8 and 24), 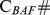
7 (3, 15, 30, 16), 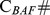
8 (31), 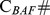
9 (23) and 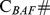
10 (1). Color bars (to the right of Figures 3A, 3B) indicate: visual (red), somatomotor (orange), auditory (green), left, right and bilateral RSNs (red) and as in Table in Fig. 2C, RSNs of intermediate region are in (black). Scale bars represent the number of shared voxels.

We computed the overlap matrix between the 30 RSNs and the Brodmann Areas (BAs) and, in order to limit the size of the matrix, we then regrouped small adjacent BAs, thereby obtaining 28 extended BAs (see Fig. 3A).

We applied the same clustering algorithm as described above, to the RSN×BA matrix. Fig. 3A displays the RSN×BA matrix, where RSNs along the vertical axis and BAs along the horizontal axis are sorted according to the results from the cluster analysis. Eight clusters were obtained (see the legend of Fig. 3A). There were three RSNs clusters that remained localized and appeared on the upper part of the matrix diagonal. These three clusters corresponded to visual (BA# 17, 18, 19), somatomotor (BA# 2, 3, 4, 5, 6) and auditory (BA# 42, 43, 22, 21) areas. The lower part of the matrix including 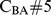, 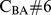 and 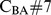 corresponded to associative-parietal (40,39), temporal areas (20,21) and all frontal and cingulate areas.

**Figure 4:**
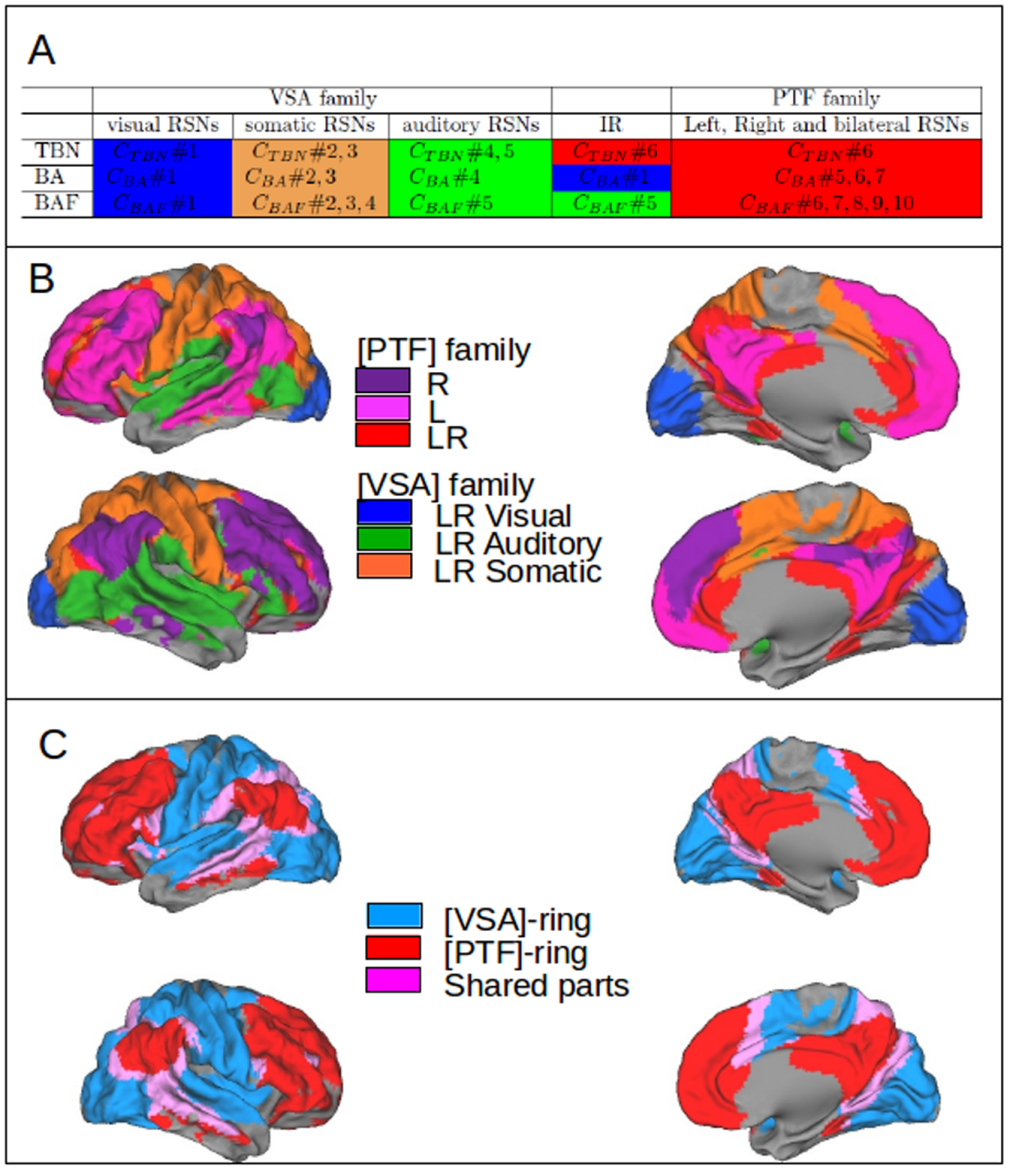
Mapping of VSA and PTF families. (A) Comparison of clustering results on the 3 matrices 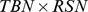, 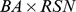 and 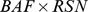. All clustering results are obtained with the same method: Mixture Distribution Algorithm. 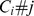 is a cluster of RSNs obtained from matrix (RSNxi), 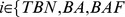, 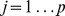, where p is the number of clusters. IR: Intermediate Regions are represented by RSN# 1, 23, 27 (B) Mapping of the 2 families which group the RSNs, according to the clustering based on functional specialization (as shown in Fig. 2) and the clustering based on cortical topography (shown in Fig. 3), giving both the same results: VSA family formed by 3 clusters, LR (Left and Right = bilateral) Visual RSNs, in blue; LR Auditory RSNs in green and LR somatomotor RSNs in orange, with similar colors as in Table in Fig. 2C. PTF family: R (right lateralized) RSNs in dark purple, L (left lateralized) RSNs in light purple and LR (bilateral) RSNs in red, distributed over the parietal, frontal, temporal and cingular regions. (C) The two intertwined rings per hemisphere: the visual, somatomotor and auditory RSNs clusters shown in Fig. 4 and grouped in the VSA family form the VSA ring, in blue, and the RSNs of the PTF family, distributed over the parietal, temporal, frontal and cingular regions form the PTF ring, in red. The overlap between the two families is shown in purple. The two rings are intertwined as shown in Fig. 7.

The results obtained for the RSNs and BAs were very close to those obtained for the RSNs and TBNs (see Table in Fig. 4A). The 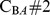 and 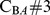 correspond to the 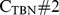 and 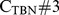 somatic clusters and the 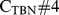 corresponds to the 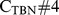 and 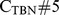 auditory clusters. Finally, the 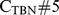, 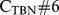 and 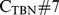 correspond to 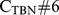 (large association networks). For a cluster 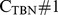, in addition to RSN# 2, 22, 5 and 9 included in the visual 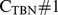 cluster, two other RSN# 23 and 1, were associated with this cluster. RSN# 23 and 1 were balanced between the two clusters: 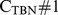 and 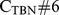.

In order to confirm the topographical organization of RSNs given by the RSN×BA matrix, we regrouped the BAs into large continuous regions. We call these regions the Brodmann Area Families (BAFs), based on their anatomo-functional interpretation: occipital visual regions BAF7 (BA# 17, 18, 19), central somatomotor BAF6 (BA# 2 until 6), temporal auditory BAF5 (BA# 41, 42, 21, 22, 37), cingular BAF4 (BA# 23 posterior and 32 anterior), inferior frontal BAF3 (BA# 44, 45, 47), superior frontal BAF2 (respectively BA# 8, 9 and 46), and anterior frontal BAF1 (BA# 10 and 11). These results are shown in the BAF×RSN overlap matrix in Fig. 3B.

Ten clusters were obtained by applying the Mixture Distribution Algorithm (see Fig. 3B). The results obtained for the RSNs and BAFs were very close to those obtained for the clustering based on the BAs overlap and the TBNs overlap. 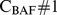 corresponds to visual clusters (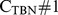), 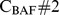, 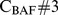 and 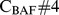 correspond to somatic clusters 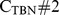 and 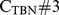 and 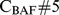 corresponds to the auditory clusters 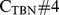 and 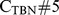. Finally, all the RSNs of clusters 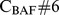, 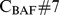, 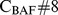, 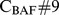 and 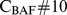 correspond to 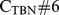, with the exception of RSN# 27, which was balanced between the two clusters, 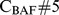 and 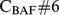.

The results obtained using a second clustering method [24] show the same distribution as the first clustering method. The similarity significance for the results obtained from RSN×BA is 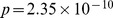 and from RSN×BAF is 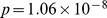.

### Corticotopy of the RSNs clusters: the dual intertwined rings architecture

The various approaches used to analyze the relationships between the RSNs, TBNs and BAs (see Table in Fig. 4A) thus all converge to a pattern which suggests that the RSNs can be grouped into two families characterized by their topography and their functions.

Twenty-seven of the 30 RSNs were specialized within one of the two families. Three RSNs (27, 1, and 23) had more balanced overlaps with the two families.

**Figure 5:**
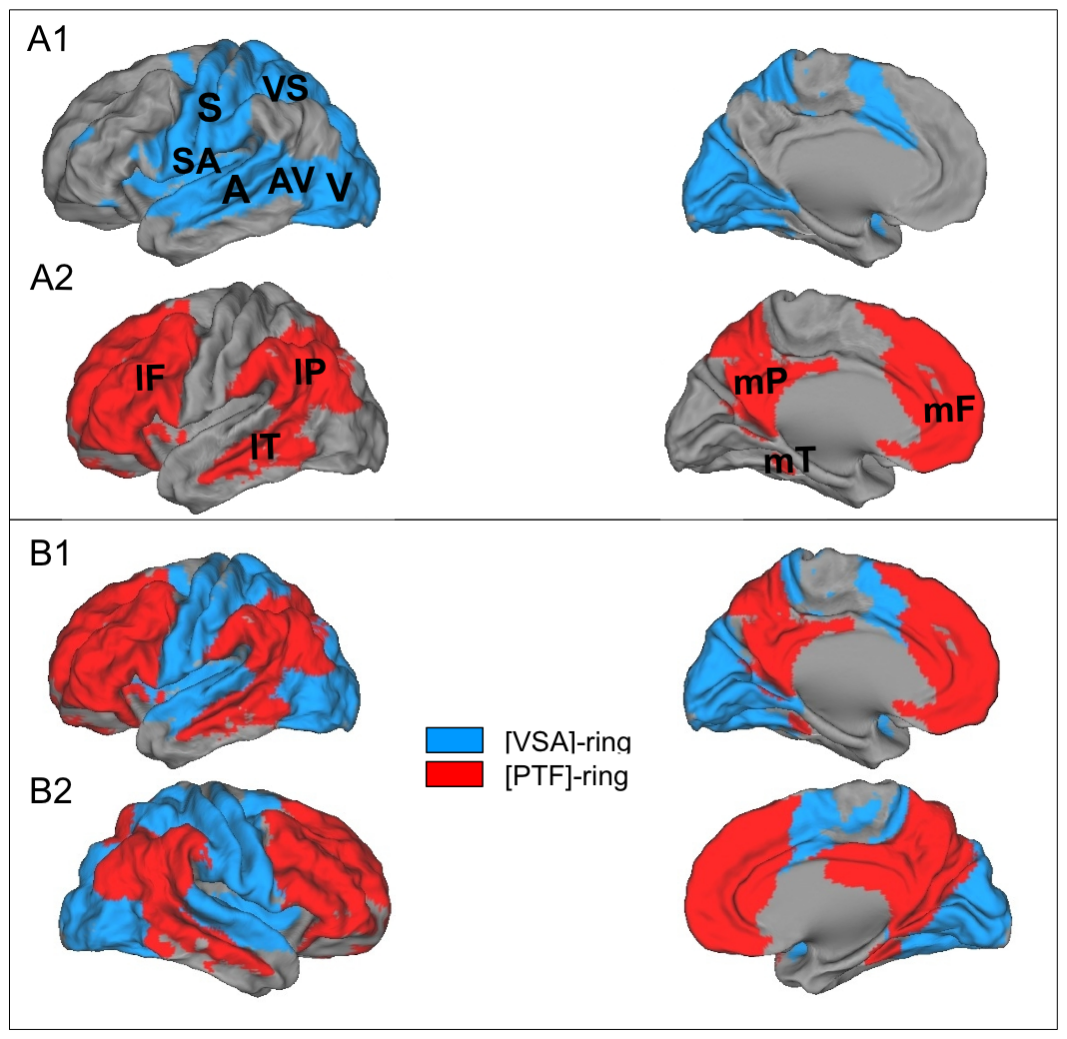
The dual intertwined rings architecture. (A1) The VSA ring, in blue, forms a continuous cortical ring organized around primary cortices: visual (V), auditory (A) and somatomotor (S) with interspersed bimodal regions: visuo-somatomotor (VS), auditory-somatomotor (SA) and visuo-auditory (VA). (A2) The PTF ring, in red, forms a ring discontinuous over the cortical mantle but closed by major cortical fiber tracts (see Fig. 6), with 3 regions, parietal, temporal and frontal on the lateral (l) aspect of each hemisphere (lP,lT,lF) and 3 regions parietal, temporal and frontal, on the medial (m) aspect (mP,mT,mF). (B) The two rings are intertwined: the PTF ring, in red, is placed in foreground, to show that it is not continuous over the cortical mantle but interrupted by the VSA ring and is closed by major cortical fiber tracts passing below the VSA ring, as shown in Fig. 6.

The first family comprises three contiguous clusters which share similar anatomo-functional principles (see Tables in Fig. 2C and Fig. 4A): The visual cluster overlaps with TBNs specialized for vision: not only with low-level vision in primary areas, but also to higher level vision in secondary areas. The somatomotor not only cluster overlaps with TBNs specialized for tactile and motor functions, but also with bimodal functions, visuo-motor and auditory-motor. The auditory cluster overlaps not only with primary and secondary regions, but also with regions involved in speech processing and bimodal auditory-visual integration. We call this set of clusters the visual-somatomotor-auditory (VSA) family of RSNs. We then mapped this VSA family, first with 3 colors corresponding to the 3 clusters (Fig. 4B) and then with a unique color to show the topography of the whole VSA family (Fig. 5A).

The second family comprises RSNs that are distributed over several, not fully contiguous, association cortices in the parietal, temporal, and frontal regions. Because this family encompasses a large set of cognitive functions distributed over the parietal, frontal, and temporal regions (see Table Fig. 2C), we call it the PTF family. Table in Fig. 2C shows that RSNs in this family overlap (1) with bilateral TBNs specialized for self-referential and social interactions (DMN); (2) with bilateral TBNs specialized for biological regulations, olfaction, taste, interoception and reward;(3) with left-lateralized TBNs specialized for language and (4) with right-lateralized TBNs specialized for working memory and attention. We then mapped the PTF family first by showing the 3 clusters, bilateral and lateralized, in 3 different colors (see Fig. 4B) and then with a unique color to show the topography of the whole PTF family, as shown in Fig. 5A2.

**Figure 6:**
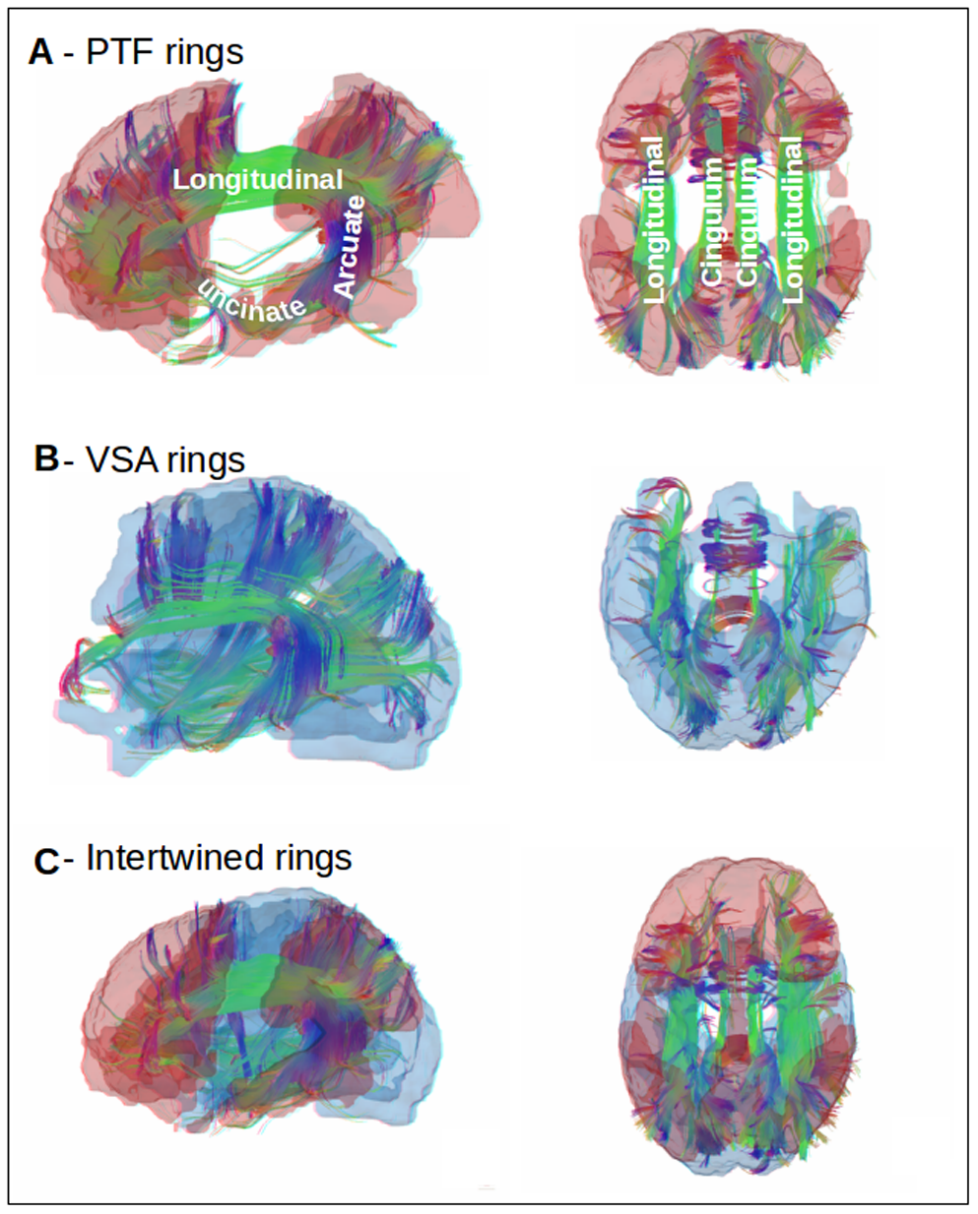
Long distance cortical connections closing the PTF ring. Comparison of the topography of the two rings (lateral and dorsal views), with superimposed major cortical fiber tracts (see text for details). Mapping of major long-distance fiber tracts on the 3D mask of the PTF ring (Fig. A) and VSA ring (Fig. B). Long-range connections on the VSA ring and the PTF ring, mapped together (Fig. C).

**Figure 7:**
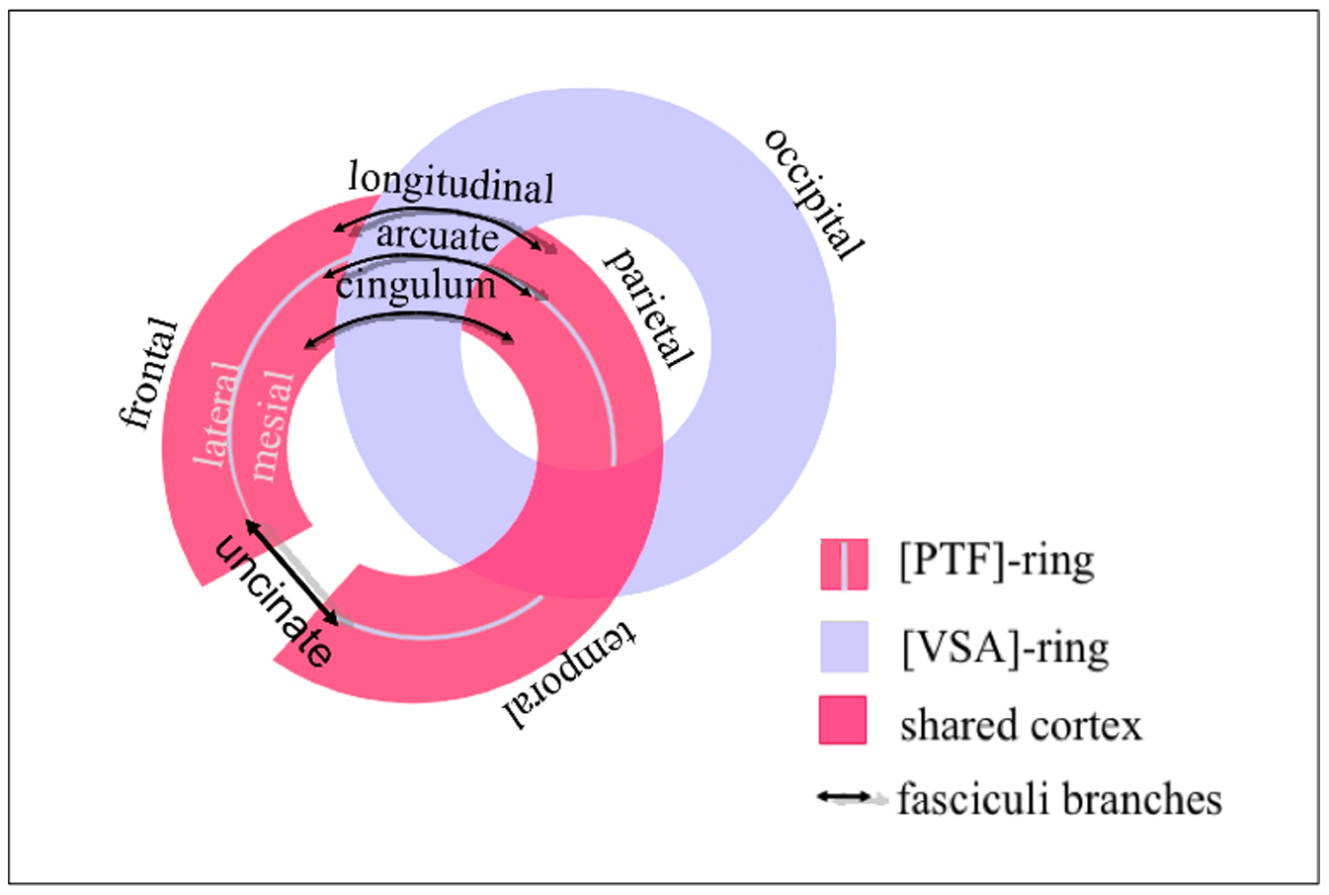
Intertwining scheme. Schematic representation of the principle of intertwining of the VSA ring and the PTF ring within each hemisphere, thanks to the major long-range tract fibers.

When the VSA and PTF families are mapped together onto the cortical surface (Fig. 4B and Fig. 4C) or displayed independently (in Fig. 5A), the mapping results reveal a remarkable 3-D structure forming two intertwined rings per hemisphere and so four rings linked by homotopic connections. The ring formed by the VSA family in each hemisphere is continuous, as shown in Fig. 5A1. Conversely, the ring formed by the PTF family in each hemisphere is discontinuous over the cortical mantle (see Fig. 5A2), but is closed by the major cortical fiber tracts, as shown in Fig. 6A. The topological model of the two rings within each hemisphere is shown in Fig. 7. We call these two rings the VSA and the PTF rings. The 3D rings extend both on the lateral and on the medial face of each hemisphere. The two families in both hemispheres are thus forming pairs of VSA rings and pairs of PTF rings. The homologous rings within each pair are symmetrical as shown in Fig. 5B. All the RSNs of the VSA family are bilateral and extend in homologous regions of the left and right VSA rings, with a very accurate symmetrical organization between the two hemispheres. The RSNs of PTF family are either bilateral and extend on the left and right PTF rings or lateralized and restricted to one ring, corresponding to the right-lateralized TBNs for attention and working memory and the left-lateralized TBNs for language.

The VSA ring (Fig. 5A1) forms a continuous cortical ring, with several sectors organized around sensory and motor maps (visual, auditory, and somatomotor), with unimodal hierarchical cortices around these primary maps and interspersed bimodal associative regions (visuomotor, auditory-visual, and auditory-motor): VS for bimodal visual and somatomotor processing, SA for bimodal auditory and somatomotor processing, related to tongue control and important for speech and VA for bimodal visuo-auditory integration. These different regions are radially distributed and bimodal sectors are interspersed between unimodal sectors. Thus, the VSA ring forms a continuous ring with six alternating sectors, monomodal and bimodal sectors. The VSA ring circles a central inferior parietal zone, which does not belong to the VSA ring but to the PTF ring (see below) and corresponds to BA# 39 and 40. This central position suggests that this sector of the PTF ring might receive, transform, and integrate all combinations of monomodal and bimodal information computed by the VSA ring. The VSA ring extends on the medial side, with two medial sectors in direct continuity with the lateral sectors. The shape of the ring is related to the topographical organization of four major cortical sulci: the calcarine sulcus (visual), the intraparietal sulcus (visuomotor), the central sulcus (somatomotor), and the superior temporal sulcus (auditory and auditory-visual).

The PTF ring (Fig. 5A2) comprises the parietal, temporal, and frontal regions, including cingular regions, that, together, form a second ring. This ring is not entirely continuous over the cortical mantle, but features ring closure, as soon as one considers the fiber tract systems that massively and directly connects cortical components of the family which are not contiguous over the cortical mantle (see next paragraph). Fig. 5A2 shows three sectors of this ring on the lateral aspect of each hemisphere and three sectors on the medial aspect. (1) The lateral parietal sector (lP) consists of supramodal parietal areas BA# 39 and 40; (2) the lateral temporal sector (lT) consists of supramodal temporal areas BA# 20 and 21; (3) the lateral frontal sector (lF) consists of supramodal frontal regions, in the IFG (BA# 44, 45, 47) and the SFG (BA# 8, 9, 46), as well as in frontopolar regions (BA#10); (4) The medial frontal sector, (mF), consists of the anterior cingulate cortex, the anterior medial prefrontal cortex and the orbitofrontal cortex (BA# 11); (5) the medial parietal sector (mP) consists of the precuneus and posterior cingulate cortex; and (6) the ring can be extrapolated to the medial temporal sector (mT) containing the medio-temporal and parahippocampal regions. The lateral and medial aspects of the PTF ring can be superimposed.

We describe the VSA and PTF rings within each hemisphere as being “intertwined,” (see Fig. 5B), because associative parietal regions of the PTF ring are at the center of the VSA ring and are connected with other regions of the PTF ring via long-range association tracts passing under the VSA ring, as shown in Fig. 6. In the following, using diffusion weighted imaging (DWI) results, we verified that the topography of the well-known long-range fiber tracts connect discontinuous regions of the PTF ring and close this ring.

#### Anatomical connections within and between the dual rings

We compared the topography of the two RSNs families with the underlying cortical fiber tract systems (see Method section). To do so, we computed the 3D masks corresponding to each RSN family, as shown in Fig. 6 and identified the long-distance fiber tracts specifically associated with each family (see Fig. 6A for the PTF ring and Fig. 6B for the VSA ring). The PTF ring presents discontinuities at the level of the cortical mantle (Fig. 6A). Actually the PTF ring is topographically cut by the VSA ring, isolates, like an island, the inferoparietal sector of the PTF ring from the rest of the ring in the middle of the VSA ring. However, the tractography results revealed that long distance association tracts constitute the structural backbone of the topological continuity of the PTF ring (Fig. 6A). The lateral parietal and frontal sectors are connected by parieto-frontal connections, formed by the inferior and superior longitudinal fasciculi. The lateral temporal, lateral parietal and lateral frontal sectors are connected by the arcuate fasciculus. The medial frontal and parietal sectors (including the anterior and posterior cingulate regions) are connected by the cingulum. Finally, the anterior lateral frontal and temporal regions are connected via the uncinate fasciculus. It can therefore be considered that the PTF ring is “intertwined” with the VSA ring and is complete, thanks to the long distance cortical connections passing below the VSA ring. The backbone of the connections underlying the VSA ring is quite different in its organization from that of the long distance cortical connections underlying the PTF ring. Long distance VSA ring connections are mainly cortico-subcortical, rather than cortico-cortical and connect the cortex to the sub-cortical structure via major projection fiber tract systems, in accordance with the sensorimotor specialization of the VSA ring. No obvious major structural differences appear between the short-range connections intrinsic to the two rings. The 3D mapping of the relationships between the two RSNs families and the infrastructure of long-range connectivity reveals the manner in which these two rings are intertwined, thanks to these connections (Fig. 6 and schematic representation in Fig. 7).

## Discussion

We investigated the large-scale topographical organization of cortical RSNs networks, and we analyzed their correlations with TBNs, BAs, and fiber tracts between areas. We extracted the 32 most representative RSNs in a large group of subjects and verified that these RSNs were the same in another independent group. Then we investigated the RSNs topographical organization by measuring their overlap with BAs and inferred their functional specialization by measuring their overlap with TBNs. We used this approach because the TBNs extracted from large databases and the BAs are well-defined, independently of the RSNs. The overlap between RSNs and TBNs (provided by the RSN×TBN matrix) revealed clusters that correspond to the major specializations of visual, auditory, somatomotor, and also bimodal functions. It also showed a large cluster that encompasses all higher cognitive functions, as well as emotions and basic needs. The overlap between the RSNs and BAs (RSN×BA matrix) revealed RSN clusters, which link (1) adjacent cortical regions (e.g. BA#17 with BA#18) and (2) other clusters that link distant regions (e.g., parietal BA#39 with frontal BA#46). Furthermore, the results showed that both properties are closely related. RSNs clusters having the same topography as the sensorimotor and bimodal regions (VSA family: visual-somatomotor-auditory), link adjacent areas. RSNs clusters having the same topography as the TBNs corresponding to higher cognitive functions, emotions and basic needs, also link distant cortical regions: parietal, frontal, temporal, and cingulate (PTF family: parieto-temporo-frontal). These highly significant results are also robust because similar results are obtained with different clustering methods (see [24], [25]).

The topography of these two families of RSNs over the cortical mantle is quite remarkable and can be described by two intertwined rings per hemisphere and so four rings linked by homotopic connections. The VSA family forms a continuous ring, in which the three bimodal functions are interspersed between the three monomodal regions. The PTF family encompasses parietal, temporal, frontal, and cingulate regions and it forms a second ring, which is discontinuous over the cortical mantle but closed through systems of long-range fiber tracts, both laterally and on the mesial wall. We described the architecture of the PTF and VSA rings as “intertwined” because a subset of associative parietal regions of the PTF ring is at the center of the VSA ring and these regions are connected with other regions of the PTF ring via long-range association tracts passing under the VSA ring. Using tractography from DWI, we verified that the topography of the corresponding well-known long-range fiber tracts connected these discontinuous regions of the PTF ring.

### Method of RSNs extraction and RSNs coverage

We used the sICA method [26] on a large group of subjects (198 healthy subjects) to extract RSNs. Because it computes the individual sICA decomposition, prior to clustering of similar effects across subjects, NEDICA (see Methods section) can account for possible high inter-subject variability. Moreover, with the criteria we proposed, in order to automatically define the classes from the similarity tree, we could control the relevance of these classes with respect to individual sICA decompositions. When considering highly representative classes as was done in the present study the number and spatial distributions of the resulting functional networks are similar to those extracted with other group ICA techniques. We selected a large number of components (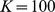) in order to identify even small networks having a high functional specificity. We found that 32 RSNs were representative and robust across subjects. We compared the RSNs from two different groups (Cambridge and Beijing) and confirmed that the RSNs with the highest representativeness had similar cortical topographies in both groups. Several parts of the cortex are not covered by the 32 RSNs: the temporal pole, some infero-temporal regions, and a part of the mesial paracentral lobule. The RSNs overlapping with these regions had a low representativeness and greater variability in the groups and did not appear among the first 32 RSNs that were retained for further analysis. Nevertheless the dual intertwined rings architecture can be easily extrapolated to these missing regions. The paracentral region encompasses the somatomotor TBNs in the VSA ring. The infero-temporal regions encompass the TBNs, in the PTF ring, associated with higher cognitive functions, language and, memory. This conjecture will have to be validated by future studies, using methods that can extend the coverage of extracted RSNs.

Likewise, it would be of interest to consider the relationships between the dual ring architecture and subcortical systems. Since subcortical-cortical pathways have a topographical organization (e.g., thalamic projections), we can predict that the VSA and PTF rings should project towards separate regions of the thalamus, basal ganglia, and cerebellum. We have limited our analysis to the cerebral cortex for two reasons. First, subcortical TBNs were not available in the TBNs database. Second, the RSNs extracted from subcortical structures were not robustly linked to regions of the cerebral cortex and were not included in the analysis. These two limitations might be overcome by using new tools for the extraction of cortico-subcortical RSNs and by extending the TBNs database to subcortical structures.

### Comparison of the dual intertwined architecture with other RSNs results

Although the remarkable organization of intertwined rings, has not been previously discussed as such, most published results dealing with the organization of RSNs present patterns that are compatible with this architecture. RSNs reflect intrinsic functional correlations between the activities of different regions of the brain and a variety of methods have been used for RSNs identification [6], [9], [13], [17]–[20], [27]–[30]. Different methods produced similar RSNs, with differences that partly depend on the pre-defined number of RSNs covering the cortical mantle, since the RSNs can be extracted at different scales.

Similarly to the findings of several other studies, the RSNs of the VSA family accurately matched the sensorimotor cortices, with different RSNs corresponding either to the central visual areas or to the peripheral areas and with the RSNs in the motor areas associated with either the hand or the mouth [8].

Other RSNs of the VSA family covered intermediate, adjacent bimodal regions forming bridges between primary unimodal areas [14]: between visual and motor areas (e.g., for visually-guided actions: grasping, reaching), between somatomotor and auditory areas (e.g., for phonation and speech) and between visual and auditory areas (e.g., for naming pictures).

The VSA ring can be described with three sensorimotor regions corresponding to primary and higher order unimodal areas and three interspersed bimodal regions. The circular structure of the VSA ring can be compared to the results of the method called “stepwise functional connectivity” [31]: when starting from primary sensory areas, RSNs correlations first arise in adjacent bimodal areas and extend to the VSA ring, and only after to the PTF ring. The global recruitment of the PTF ring is consistent with studies that have revealed a high degree of intrinsic functional connectivity between high order associative regions of the parietal, frontal, temporal, and cingular cortices [4].

RSNs in the PTF family correspond to RSNs classically reported by other studies, such as the highly reproducible DMN network, together with similar RSNs that correspond to lateral fronto-parietal connections in the PTF ring, the fronto-parietal control network, the dorsal attentional network and the ventral attentional network [5], [10], [16]–[22], [28]. Our classification into two families, based on overlaps with BAs and TBNs, confirmed previous works searching for hierarchy of RSN cluster obtained when extracting clusters using only the resting state data [8], [12], [15], [31], [32].

Several methods (e.g., hierarchical cluster analysis [12], Gaussian Bayesian network [32] and fuzzy-c-means clustering algorithms [15]) gave results consistent with a two cluster organization: the first cluster comprising visual areas and primary somatosensory and auditory areas, and the second one comprising the default mode, fronto-parietal, dorsal attentional, and ventral attentional networks. In our study we show that two similar families can be obtained also by a different method measuring overlaps between RSNs, TBNs, and BAs. However, here we structure the topography of these two families of RSNs into intertwined rings and characterize their functional specialization in relation with two sets of TBNs.

Long distance cortico-cortical connections are essential to support a dual intertwined ring architecture. When the ring topography is visually compared with the main cortico-cortical fiber tract systems [33], the geometrical similarities between the dual ring architecture and the long-range fiber tracts is striking. Furthermore, using tractography from DWI data in three subjects, we controlled that the pattern of long-range connectivity of the PTF ring well matched its geometry and completed its closure. The dual intertwined architecture is thus consistent with studies that have demonstrated significant correlations between structural and functional connectivity, even if RSNs can also correspond to polysynaptic pathways [22], [34]–[36].

In previous reports, RSNs corresponding to the PTF ring have often been broken down into two subsets of networks called task-positive and task-negative systems [5]. The task-negative network (i.e., the default mode network), shows a decrease in activity during task performance [10], [37]–[41] while the task-positive network is activated during goal-directed and cognitively demanding tasks. In these experiments, the task concerns executive functions, while regions of DMN activation are more active in internal tasks related to self, memory recall and internal thoughts. In the intertwined ring architecture, this dichotomy can be related to the topography of the PTF ring. On the mesial wall, the ring relates the DMN network to regions of the anterior part of the ring that are important for internal functions of biological regulation and biological rhythms. On the lateral part of the PTF ring, the intertwining relates the parieto-frontal network of the PTF ring (executive functions) to the VSA ring essential for integration of interactions with the external world.

As defined in this paper, the topography of the rings and their components are stable even though the RSNs, composing the rings, can vary because their identifications vary with the methods, the parameters [8], or even they are dynamic and may change over time [42], [43]. The rings are stable because they are defined as sets of well-defined BAs and robust statistically defined TBNs [14], [44]. The dual intertwined architecture can be viewed as an organizational scheme describing the changing topography of the flexible RSNs within the fixed architecture of the two rings. Similarly, task related activations in individual experiments are highly flexible, reflecting the variety of tasks and experimental conditions. Recent studies have directly compared the resting state networks (intrinsic networks) and the networks activated during a set of tasks (extrinsic networks) at the participant level [45]. These results demonstrated that there is a weak correspondence between individual TBN and RSN, for RSNs that we have classified in the VSA ring and a strong correspondence for RSNs that we have classified in the PTF ring. In the framework of the dual ring architecture, these results suggest that individual TBN tend to activate specific parts of the VSA ring, spreading over local functional links, while activations spread in a more extended over parts of the PTF ring, through long-range connections.

### Real-time integration in the VSA ring and multi-temporal integration in the PTF ring

The dual intertwined architecture seems to underline also a dual integrative process: the VSA ring performs fast real-time multimodal integration of sensorimotor information, to control interactions with the environment while the PTF ring performs multi-temporal integrations (i.e., relating past, present, and future representations at different temporal scales), as described below. Sensorimotor processing in the VSA ring implies strong “real time” constraints, designed to link various sources of auditory, visual and somatomotor information together and to control actual behavior. These real-time interactions are important, not only within each modality as sensory or motor, but also for all of their bimodal interactions: between visual and motor (e.g., grasping, reaching, imitation) between auditory and somatomotor information (e.g., recognizing and producing phonemes) and between auditory and visual information (important for communication).

Our results show that the PTF ring implements at least four main groups of functions (TBNs, Table in Fig. 2C): (1) biological regulation, olfaction, taste, and emotion, (2) working memory and attention, (3) self-referential functions and social cognition and (4) language. The common neural processes supporting these functions (in contrast to sensorimotor real-time processes,) integrate different sources of information in a “multi-temporal” fashion, at different time-scales, including recall from the past and projections into the future.

Biological regulations and rhythms (in the medial and anterior part of the PTF ring: TBN#2,4 in [14]), operate at different time scales, and are strongly influenced by (1) the hypothalamus and hormonal regulations ranging from minutes to days and months and by (2) the dopaminergic system involved in drive, reward and reinforcement, ranging from minutes to hours. Similarly, emotions are prolonged reactions, which last much longer than the induced emotional sensory stimuli: olfaction and taste produce neural effects which last much longer than those produced by real-time vision, hearing, or touch.

Planning and working memory (in the lateral frontal network of the PTF ring) are based on the integration of information across time by sustained neuronal activations and are able to integrate in the same sequence, several sensory and motor events separated by long and variable delays (review in [46]–[51]).

Self-referential functions (in the medial fronto-parietal part of the PTF ring) require information to be integrated at different temporal scales from the past (memory), the present and the future [4], [38], [41], [52]–[55]. These regions are active both when subjects consider their present mental states and when they make inferences about the mental states of others (theory of mind and social cognition) [56]–[59]. Such processes can last for minutes to hours.

Language processing (left temporo-parieto-frontal part of the PTF ring) requires the formation of meaningful conceptual representations integrating events and their consequences over long time lines. It is striking to observe that the regions in the PTF ring match regions that are more strongly activated for words having a meaning than for pseudo-words, which, by contrast, are processed only in the VSA ring [11]. The PTF ring has a topography quite similar to brain activations related to semantic processing [10], [11], [60]. In contrast, speech processing requires real-time processing of visual form perception, motor articulation and auditory perception and, accordingly, the corresponding TBNs are in the VSA ring.

The dual process that we postulate across the two rings (i.e., real-time and multi-temporal integration), are close to the dual process proposed by Fuster [61], [62] with both perception-action cycles and working memory (WM) processes. A perception-action cycle requires temporal contiguity between different signals. By contrast, WM based on sustained neuronal activation is critical to the integration of information across time in goal-directed behavior, reasoning and language bridging time in the perception/action cycle. WM has a retrospective function of retention and a prospective function of anticipation and preparation for forthcoming actions. It allows the temporal structures of strategies, melodies, sentences, scripts, etc. to be stored in memory [61], [62]. Furthermore, the WM processes depend on interactions between large-scale networks of the cerebral cortex, involving the co-activation of several non-contiguous cortical areas (e.g., a lateral prefrontal region and, concomitantly, a region of the posterior cortex).

### Topological advantages of the intertwining

As an important topological advantage, the dual intertwined rings architecture creates multiple interfaces between the two rings within each hemisphere for a large variety of cognitive and sensorimotor functions. First, the intertwined architecture forms three large interfaces between the two rings, which correspond to three large sulci: the precentral sulcus, the STS sulcus, and the intraparietal sulcus. The precentral sulcus forms a VSA-PTF interface for tasks associating multi-temporal goals, and subgoals (frontal areas) with real-time command of each action (somatomotor areas). The STS sulcus forms a second VSA-PTF interface for tasks associating real-time speech (superior temporal areas) with the multi-temporal signification of words (inferior temporal areas). The intra-parietal sulcus forms a third VSA-PTF interface for tasks involving the real-time visuomotor processing of scenes and events and multi-temporal processing of these scenes in terms of social interactions. Second, the intertwining architecture places the associative parietal areas BA#39 and 40 (which are part of the PTF ring) in the central hole of the VSA ring at the confluence of visual, spatial, somatosensory and auditory processing streams (see [63]). This same BA39 region is also related to the medial part of the PTF ring that is implied in the representations of intentions and social interactions. The BA39 can thus transform the real time observation of actions, scenes, and events processed in the VSA ring, in longer term predictions and interpretations related to intentions and social interactions processed in the PTF ring (see [64], [65]). Third, the intertwined architecture places the VSA somatomotor regions in a central position between the PTF parietal and frontal areas, which are themselves connected by the parieto-frontal network. Somato-motor regions can command actions depending both on goals and subgoals (links with frontal regions) and the interpretation of scenes (links with parietal regions).

### Development of the dual intertwined rings architecture

A significant finding that emerged when measuring RSNs in children is that developing RSNs are quite similar to the adults' RSNs [66]–[70] and it is even possible to detect these RSNs in preterm infants [71], [72]. Interactions progressively change from being predominately anatomically local in young children, to interactions spanning longer cortical distances in young adults, with the segregation of local regions and the integration of distant regions [72]–[75] (but this result should be confirmed to exclude possible motion artefact [76], [77]). The distributed cerebral networks of the association cortex have a late development in terms of myelination and cortical surface area [78], thereby facilitating increased long-range correlations. In the dual ring framework, this suggests that the VSA and the PTF rings are present at birth. The RSNs of the VSA ring mature first and can facilitate the fast development of sensorimotor functions during the first years. The RSNs of the PTF ring mature more slowly and can facilitate the further development of higher cognition. Stronger functional inter-regional connectivity mediates higher level control functions, leading to a more mature cognition.

### Evolution of the dual intertwined ring architecture

Previous results comparing monkey and human brains strongly suggest an expansion in humans of the associative parietal, temporal, and frontal areas, with greater functional specialization [78]–[80]. In humans, the inferior parietal lobule includes two Brodmann cytoarchitectonic areas that are absent in the monkey (areas 39 and 40). Long-distance networks exist in monkeys, with a functional equivalent of the human DMN [36], [81]–[83], but monkeys lack the lateralized fronto-parietal RSNs implicated in language functions [36], [83], [84]. In the dual ring framework, these results strongly suggest the development of the PTF ring during evolution, but also the parallel development of the intertwined architecture, with an expansion of parietal areas of the PTF ring in the center of the VSA ring, connected both to lateral fronto-temporal and medial parieto-prefrontal regions. This architectural evolution could be important for human language, because it could relate the processing of words and sentences in the temporo-frontal network with the interpretation of scenes and social interactions in the medial parieto-prefrontal regions.

### Conclusion

To conclude, because the dual intertwined rings architecture integrates large set of anatomical, functional, and cognitive results, it can broaden our understanding of the functional properties of the cerebral cortex and the corticotopy of sensorimotor and cognitive functions. In turn, the functional interpretation of the dual ring architecture (real time vs. multi-temporal integration) could inspire new models of the neural basis of these sensorimotor and cognitive functions.

## Materials and Methods

### Resting-state database

Resting-state fMRI data were acquired from 198 healthy volunteers (age: 18–30) (from FCP Classic-Cambridge,). Another similar database comprising 170 healthy volunteers (age: 18–26) (from INDI Beijing enhanced) was used to confirm the results obtained from the first onset of volunteers. Both databases are freely available from the NITRC (http://fcon_1000.projects.nitrc.org) [85]–[87]. Acquisition parameters for the Cambridge databases were: TR = 3000 ms, 47 contiguous slices, 119 volumes, matrix size = 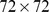, voxel size = 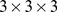
mm^3^; and for the Beijing databases were: TR = 2000 ms, 33 contiguous slices, 225 volumes, matrix size = 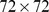, voxel size = 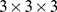
mm^3^; For each individual in both populations, a T1-weighted sagittal MP-RAGE structural image was obtained from the same databases.

### Pre-processing

Individual time-series were corrected for slice-timing, within run motion and smoothed (FWHM = 5 mm) using the FSL software (http://www.fmrib.ox.ac.uk/fsl/). A nonlinear transformation, used to register the fMRI volumes into the MNI standard space, was calculated as the combination of a linear transformation (using FLIRT), between the averaged fMRI volume and the T1 volume in the subject space and a nonlinear transformation (using FNIRT) was used between the T1 volume in the individual space and the T1 template in the MNI standard space. Then, the FA and ADC images were registered on a 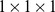
mm^3^; standard space image (MNI152 space), using a nonlinear registration procedure.

### Functional network identification by spatial Independent Component Analysis (NEDICA)

Group functional networks were identified using a two-step procedure, the NEDICA method [26]. The first step consisted in applying a spatial independent component analysis (sICA) to each individual fMRI-series, with a fixed number of components 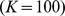. The second step consisted in identifying reproducible spatial components across subjects, by means of hierarchical clustering. RSNs were then identified according to their representativeness in the studied population R(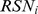): the number of subjects in which 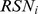 is present was divided by the total number of subjects (among group-representative clusters identified for at least 10% of the subjects). Finally, the corresponding T-maps were thresholded at 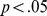, with a false discovery rate procedure used as a control for multiple comparisons.

### Clustering methods

The aim of this work is to build methods for sample classification and selection of RSNs networks in order to identify the various functional and anatomical RSNs groups. Our method is based on a Bayesian approach to density estimation and clustering using mixture distributions [25]. For our data sets comprising multinomial variables (values overlap between RSNs and TBNs, BAs or BAFs), we used the approach involving mixture distributions having Gaussian components fitted by maximum likelihood, for which the EM algorithm has a closed-form. This algorithm allows the automatic determination of the number of distributions in the mixture. One of the most common motivations for using Gaussian distributions is to obtain robust estimates for the RSNs groups. To evaluate the accuracy of Bayesian classification, we compared this technique with the community structure detection algorithm [24] to evaluate if the results of the two methods agreed sufficiently closely. Indeed, if we consider that each data set is a network overlap, the community structure method assumes that the network of interest divides naturally into subgroups. The number and size of the groups are thus determined by the network itself. The true community structure or group can be quantified by using the modularity measure [88]) which is defined as the number of edges falling within groups minus the expected number in an equivalent network with edges placed at random. The accuracy of a Bayesian classification is assessed by comparing the classification with community structure detection algorithm. The results of the accuracy assessment are summarized in a confusion matrix which displays the number of correct and incorrect predictions made by the Bayesian model compared with the community structure detection algorithm. The estimate of accuracy is then simply the ratio of correct predictions to the total number of predictions.

### RSNs specialization: overlap with TBNs

We characterized the functional properties of the 32 RSNs by computing their overlaps with the TBNs extracted from large databases of fMRI activation, during sensorimotor and cognitive tasks. This analysis was carried out using the following measurement of topological similarity:
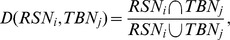
where 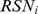 are the binary thresholded T-map of the 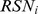 and the masks of 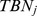 [14].

We then constructed a similarity matrix between each RSN and each TBN. Each RSN was then characterized by its overlap with the TBNs specialized in cognitive functions (see [13], [14]). In order to find RSNs clusters with similar functional specializations, the similarity matrix was reorganized by applying an Expectation Maximization (EM) algorithm as described in [25]. The reorganization of this similarity matrix provided several clusters functionally characterized by TBNs (see Fig. 2C).

### RSNs distribution: overlap with BAs regions

We characterized the anatomical organization of the 32 RSNs by computing their overlaps with Brodmann areas. The Brodmannn areas (BAs) numbered from 1 to 47 were grouped into 28 regions (the small adjacent regions were regrouped) to limit the size of the matrix (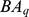, 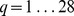):
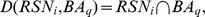
where 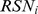 are the binary thresholded T-map of the 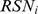 and 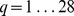. The resulting similarity matrix was reorganized by applying the same Mixture Distribution Algorithm as that described above. The RSNs clusters were then defined and characterized anatomically by the Brodmann atlas. To confirm this result and show that it does not depend on the number of BAs regions, we grouped the 28 BAs regions into 7 families (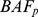, 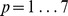), related to the occipital lobe (BA# 17, 18, 19), the central sulcus (BA# 2, 3, 4, 5, 6), the temporal lobe (BA# 41, 42, 21, 22, 37), the cingular cortex (BA# 23, 32), the inferior frontal cortex (BA# 44, 45, 47), the superior frontal cortex (BA# 8, 9, 46) and the anterior frontal cortex (BA# 10, 11). The RSNs were then anatomically localized using the spatial overlap ratio between the RSNs and the BAFs:
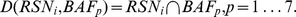
Next, we constructed a similarity matrix between each of the (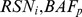) pairs, which were then reorganized by applying the same clustering method.

### Anatomical Fiber tracting

We compared the topography of RSNs, made of non-adjacent cortical regions, with underlying cortical fiber tracts. The data used in the preparation of this work were obtained from the Human Connectome Project (HCP) database https://ida.loni.ucla.edu/login.jsp. The HCP project (Principal Investigators: Bruce Rosen, M.D., Ph.D., Martinos Center at the Massachusetts General Hospital; Arthur W. Toga, Ph.D., University of California, Los Angeles, Van J. Weeden, MD, Martinos Center at the Massachusetts General Hospital) is supported by the National Institute of Dental and Craniofacial Research (NIDCR), the National Institute of Mental Health (NIMH) and the National Institute of Neurological Disorders and Stroke (NINDS). HCP is the result of contributions from co-investigators from the University of California, Los Angeles, the Martinos Center for Biomedical Imaging at Massachusetts General Hospital (MGH), the Washington University and the University of Minnesota.

A diffusion spectrum MRI scan was acquired in a normal human subject, imaged in vivo at 3 Tesla with an isotropic spatial resolution of 2 mm and 515 diffusion samples (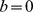 every 15 volumes) with a peak gradient intensity Gmax = 90 mT/m. Diffusion orientation density functions (ODFs) were reconstructed using the 3D discrete Fourier transform of the modulus of the measured signal. From the directions of the ODF maxima, paths were computed with a streamline tractography algorithm (parameters: Euler integration; angular threshold 35 degrees; step length 0.2 mm; 8 random seeds within voxels) and smoothed using a B-Spline filter. Next, fiber tracts were registered into the standard MNI space by means of a linear transform computed using FSL (FMRIB, Oxford, UK: www.fmrib.ox.ac.uk/fsl/). Finally, the ring masks were used to filter tracks and keep fibers which end within rings. All diffusion data processing and visualization was carried out using the TrackVis software (MGH, Massachusetts, USA: www.trackvis.org/).

### Representation of the results

The functional structures were mapped onto a standard human brain surface using the Caret software (http://brainvis.wustl.edu/).
